# A Pilot Randomised Controlled Trial Involving Financial Incentives to Facilitate Hepatitis C Treatment Uptake Among People Who Inject Drugs: ETHOS Engage Study

**DOI:** 10.3390/v16111763

**Published:** 2024-11-12

**Authors:** Alison D. Marshall, Anna Conway, Evan B. Cunningham, Heather Valerio, David Silk, Maryam Alavi, Shane Tillakeratne, Alexandra Wade, Thao Lam, Krista Zohrab, Adrian Dunlop, Craig Connelly, Victoria Cock, Carina Burns, Charles Henderson, Michael Christmass, Gregory J. Dore, Jason Grebely

**Affiliations:** 1 The Kirby Institute, UNSW Sydney, Sydney, NSW 2052, Australia; aconway@kirby.unsw.edu.au (A.C.); ecunningham@kirby.unsw.edu.au (E.B.C.); hvalerio@kirby.unsw.edu.au (H.V.); dsilk@kirby.unsw.edu.au (D.S.); stillakeratne@kirby.unsw.edu.au (S.T.); gdore@kirby.unsw.edu.au (G.J.D.); jgrebely@kirby.unsw.edu.au (J.G.); 2 Centre for Social Research in Health, UNSW Sydney, Sydney, NSW 2052, Australia; 3 Drug and Alcohol Clinical Services, Mid North Coast Local Health District, Kempsey, NSW 2440, Australia; alexandra.wade@health.nsw.gov.au; 4 Drug and Alcohol Clinical Services, Western Sydney Local Health District, Sydney, NSW 2770, Australia; thao.lam@health.nsw.gov.au; 5 Lismore Liver Clinic, Mid North Coast Local Health District, Lismore, NSW 2480, Australia; krista.zohrab@health.nsw.gov.au; 6 Drug and Alcohol Clinical Services, Hunter New England Local Health District, Newcastle, NSW 2302, Australia; adrian.dunlop@health.nsw.gov.au; 7 North Metro Community Alcohol & Drug Service, Joondalup, WA 6027, Australia; craig.connelly@mhc.wa.gov.au; 8 Drug and Alcohol Services South Australia (DASSA), Adelaide, SA 5069, Australia; victoria.cock@sa.gov.au; 9 Drug and Alcohol Clinical Services, South Western Sydney Local Health District, Sydney, NSW 2170, Australia; carina.burns@health.nsw.gov.au; 10 NSW Users and AIDS Association, Sydney, NSW 2010, Australia; 11 National Drug Research Institute, Curtin University, Perth, WA 6102, Australia; Michael.Christmass@health.wa.gov.au; 12 Next Step Community Alcohol and Drug Services, East Perth, WA 6004, Australia

**Keywords:** hepatitis C virus, financial incentive, contingency management, randomised controlled trial, treatment

## Abstract

The primary aim of this study was to establish the feasibility of implementing a larger RCT designed to evaluate the effect of financial incentives on HCV treatment initiation among persons receiving opioid agonist therapy and/or who have injected drugs in the prior six months. ETHOS Engage is an observational cohort of participants recruited from drug treatment and needle and syringe programs in Australia. Among 11 drug and alcohol clinics, participants who were HCV RNA-positive were randomized (1:1) to receive standard of care or a AUD $60 gift card at treatment initiation. Regarding feasibility, 100% (57/57) of eligible participants enrolled to take part. Twenty-eight participants were randomised to the financial incentive arm (AUD $60 gift card) plus standard of care and 29 participants to the standard of care arm. In this pilot RCT (n = 57), median age was 42 years (IQR 37–49), 63% were male (n = 36), 35% Indigenous (n = 20) and 36% (n = 21) reported injecting drugs daily in the past month. Twelve weeks post-study enrolment, 11 (39%) participants in the financial incentive arm and 17 (59%) participants in the standard of care arm initiated HCV treatment. Findings indicate high feasibility among people who inject drugs to be randomised to receive financial incentives to initiate HCV treatment.

## 1. Introduction

Worldwide, people who inject drugs are most impacted by hepatitis C virus (HCV), [1]. All-oral direct-acting antivirals (DAAs) have high cure responses (>95%), resulting in much optimism to achieve World Health Organization (WHO) targets to eliminate viral hepatitis as a public health threat by 2030 [2]. The Australian government has provided reimbursed DAAs since March 2016, but there are some people who inject drugs that have not initiated treatment [3]. Strategies to enhance linkage to HCV care among this population are critically needed.

The use of financial incentives to influence positive health behaviours (contingency management) has been extensively researched among clients of drug and alcohol treatment services, often in relation to cessation of or reduction in drug use [4,5]. There is comparatively limited research involving financial incentives and HCV-related outcomes [6]. Wohl et al. (2017) implemented a randomised controlled trial (RCT) to investigate the feasibility and effect of implementing financial incentive schemes—a fixed incentive (n = 28) or lottery-based incentive (n = 31)—on client attendance to HCV clinic visits [7]. There were no significant differences in client attendance between groups (92% attendance overall) with >90% adherence and 92% sustained virologic response 12 weeks posttreatment (SVR12). Findings from an RCT involving an HIV-HCV co-infected population—while not statistically significant—found financial incentives provided a trend to treatment uptake (45/54; 83%) compared to standard of care (24/36; 67%) [8]. There was no significant difference between groups on SVR12.

Our prior research indicated that people who inject drugs were highly willing (93%; n = 593/635) to participate in an RCT involving financial incentives to initiate HCV treatment [9]. The primary aim of this study was to establish the feasibility of implementing a larger RCT designed to evaluate the effect of financial incentives on HCV treatment initiation among persons receiving opioid agonist therapy and/or who have injected drugs in the prior six months.

## 2. Methods

ETHOS Engage is an observational cohort of participants recruited from drug treatment and needle and syringe programs in Australia [10]. Recruitment involves campaign days where participants are tested for current HCV infection, complete a computer-tablet-based questionnaire, are assessed for liver disease (transient elastography), and are linked to care. Study clinics involved in Wave 2 (November 2019–June 2021) of ETHOS Engage (n = 21) were asked if they wanted to implement the ETHOS-Contingency Management pilot study (ETHOS-CM), which included a six-item participant questionnaire about client acceptability of being randomised to participate in an RCT [9] and an RCT involving financial incentives (presented herein). Eleven clinics agreed to participate: New South Wales (n = 7), South Australia (n = 2), and Western Australia (n = 2). The primary service provided by the clinic was opioid agonist treatment (n = 9) or other drug and alcohol treatment (n = 2).

ETHOS-CM inclusion criteria were as follows: ≥18 aged years, injecting drug use in the six months preceding enrolment or reported a lifetime history of injecting drug use with current opioid agonist treatment and confirmed HCV infection (detectable HCV RNA). Exclusion criteria were current HCV treatment and pregnancy.

During the consent process for Wave 2 of ETHOS Engage, participants were informed of ETHOS-CM (six-item questionnaire and pilot RCT). Participants who declined to take part in ETHOS-CM could still participate in ETHOS Engage. Recruitment for ETHOS-CM occurred between January 2020 and June 2021. All participants in ETHOS Engage (and thus, ETHOS-CM) received AUD 30 at enrolment. Study ethics was approved by the Human Research Ethics Committees at St. Vincent’s Hospital, Sydney, and the Aboriginal Health and Medical Research Council [2019/ETH03536].

Once informed written consent was received for ETHOS-CM, participants with HCV infection were randomized (1:1) to receive standard of care (control arm) or a AUD $60 prepaid multi-store gift card at treatment initiation (intervention arm). A computerised random number generator with block randomisation (version SAS 9.4) was applied to keep participant balance between study arms. Clinic coordinators were then given a set of sealed, ordered envelopes that each contained a study arm based on the randomised list. Once HCV infection was confirmed, the clinic coordinator opened the envelope and assigned the participant to the relevant study arm. It was not possible to blind the participants, researchers, or clinic staff to the study conditions since it was highly likely that the clinic coordinator was also involved in managing HCV treatment among participants.

Given that this is a pilot RCT to assess the feasibility of randomisation in drug and alcohol treatment settings, we sought to recruit 30 participants per study arm (n = 60 total). The primary endpoint was the proportion of eligible participants who enrolled and were randomised to either the financial incentive arm (AUD 60 gift card) plus standard of care or standard of care arm (control arm) (Supplementary Figure S1). Participant feasibility was reported via a participant flow diagram and proportion of participants who completed primary and secondary endpoints. Clinic feasibility was assessed by whether clinics could recruit participants into a pilot RCT involving financial incentives to encourage HCV treatment initiation.

The secondary endpoints were the proportion of participants who initiated HCV treatment within 12 weeks of study enrolment, completed HCV treatment, and received end of treatment response, and SVR12, and time from testing to treatment initiation between study arms. Standardised case report forms were used to record the date of HCV treatment prescription (treatment initiation). Clinic coordinators reminded participants in the financial incentive arm that they would receive a AUD $60 gift card if they initiated treatment within 12 weeks of study enrolment. Per standard of care, clinic services encouraged participants to attend their treatment completion, end of treatment, and SVR12 site visits. For SVR12, intention to treat (ITT) and modified intention to treat (mITT; calculated as proportion of participants who were tested at 12 weeks post-treatment who were HCV RNA-negative) were calculated. Data were presented in percentages or median values (IQR). Chi square test was used to examine between-group differences for categorical data and Wilcoxon rank-sum test was used to examine between-group differences for continuous data. Significance was set at alpha level of *p* ≤ 0.05 and *p*-values were two-tailed. Data were analysed with STATA version 12.0 (College Station, TX, USA). Acknowledging that this is a pilot study, the sample was not powered to detect a difference in endpoints, and thus, caution was exerted when reporting implications of study findings [11].

There was one protocol violation. When the ETHOS Engage study coordinator contacted clinic sites at nearly 12 weeks post-study enrolment to retrieve data from the clinic forms, it transpired that a coordinator at one clinic had mistakenly disseminated the AUD 60 gift cards to all participants in the financial incentive arm (n = 6) regardless of whether the participants initiated HCV treatment (2/6 participants initiated treatment at this clinic). The ETHOS Engage study coordinator then re-engaged with all clinics to ensure that they understood the purpose of the pilot RCT and distinction between study arms. As a result, a sensitivity analysis excluding these six participants was conducted.

## 3. Results

Among 635 participants screened for inclusion, 93% (n = 593) were eligible (i.e., not currently receiving treatment or were pregnant) and agreed to participate in ETHOS-CM. Among those who agreed to participate (n = 593), 90% (n = 536) were HCV RNA undetectable and excluded from study participation (Supplementary Figure S2).

Regarding study feasibility among participants, 100% (57/57) of eligible participants were enrolled and individually randomised in the pilot RCT. Twenty-eight participants were randomised to the financial incentive arm (AUD $60 gift card) plus standard of care and 29 participants to the standard of care arm (Supplementary Table S1). Regarding clinic feasibility, 11 drug and alcohol treatment centres implemented the pilot RCT with all clinics able to recruit participants and most clinics (10/11) able to implement the pilot RCT without incident.

Among the 57 participants, median age was 42 years (IQR 37–49), 63% were male (n = 36), 35% Indigenous (n = 20), 89% were unemployed (n = 51), and 14% were homeless (n = 8). Thirty-six percent reported injecting drugs daily in the past month (n = 21) and 57% of participants were receiving opioid agonist treatment at enrolment (n = 33). Fifty-four percent (n = 31) had received prior treatment for HCV infection. Between arms, there were significant differences in daily injecting drug use in the last month (50% in intervention arm vs. 24% in standard of care, *p* = 0.020) and median liver stiffness (5.2 kPa in intervention arm vs. 6.7 kPa in standard of care, *p* = 0.030) (Table 1).

For the secondary endpoints, in the financial incentive arm (n = 28), 11 participants (39%) initiated HCV treatment within 12 weeks of study enrolment compared to 17 participants (59%, n = 29) in the standard of care arm (*p* = 0.144) (Supplementary Table S2). Among all participants who initiated HCV treatment (n = 28), median time to initiation was 55 days in the financial incentive arm (n = 11) and 77 days in the standard of care arm (n = 17) (*p* = 0.742). Glecaprevir-pibrentasvir (n = 20; 71%) was the most frequent DAA prescribed across both arms with remaining participants receiving sofosbuvir-velpatasvir (n = 8, 29%). There were no significant differences in treatment completion (*p* = 0.832), end of treatment response (HCV RNA-negative) (*p* = 0.634), and SVR12 (HCV RNA-negative) (*p* = 0.338) between arms (Supplementary Table S2). With regards to the sensitivity analysis excluding the six participants, there remained no difference between study arms regarding treatment initiation, treatment completion, end of treatment response or SVR12 (Supplementary Table S3).

## 4. Discussion

Findings from this pilot RCT contributed new evidence on participant and clinic feasibility of a larger RCT involving financial incentives within this cohort or another similarly pre-incentivised cohort. In this pilot sample, financial incentives (AUD $60 gift card) compared to standard of care had no significant effect on HCV treatment initiation among our study population of people who inject drugs and/or are currently receiving opioid agonist treatment. Between study arms, there were no significant differences in HCV treatment completion, end of treatment response, and SVR12.

Our prior findings demonstrated that participants were highly willing to take part in an RCT that involved financial incentives to initiate HCV treatment [9]. This study verified this sentiment, as overall, participants were willing to be randomised and retained their study participation akin to what would be expected in similar HCV treatment initiation studies [6,12]. While all 11 clinics were able to recruit participants and implement the RCT design, further research is planned with ETHOS Engage clinics who participated in this study (and chose not to participate) to better understand facilitators and barriers to implementation. We also intend to explore with stakeholders how to most optimally implement financial incentives to facilitate client involvement in HCV-related care (e.g., greater financial incentive values, use of cash instead of a gift card, and/or multiple incentives at different study visits).

With no significant effect found between study arms, findings suggest that enrolled participants may have already been sufficiently engaged in (our study’s assessment of) HCV-related care. In the design stage of a contingency management intervention, it can be challenging to delineate at which clinical endpoint a financial incentive would have the most impact. Participants in this study (and corresponding clinics) may have benefited more from client financial incentives at later endpoints (e.g., returning for SVR12 test). Further, a future RCT involving a different clinical endpoint (e.g., incentives to encourage HCV RNA testing) may be of benefit to people who inject drugs who are not currently engaged in HCV-related care. A recent systematic review and meta-analysis involving people who inject drugs and HCV-related care (46 studies) found that patient education, patient navigation, and integrated care significantly increased patient linkage to care and treatment initiation [6]. Still, there is a call for more RCTs to measure intervention impact across a range of clinics and populations so that interventions can be better tailored to clinic infrastructure and the needs of people who inject drugs [6].

We acknowledge the study limitations. The small sample size was not powered to detect a difference between the study outcomes. Every participant received a AUD 30 gift card at enrolment and the opportunity to receive a AUD $60 gift card at treatment initiation (smaller incentive versus larger incentive design). Clinics who took part in this study may have greater feasibility to implement RCTs. We did not record why participants and clinics chose not to participate. It would have also been informative to speak to participants who did not initiate HCV treatment (n = 29). One clinic (out of eleven) disseminated the AUD $60 gift card prior to the participants in the financial incentive condition initiating treatment (Supplementary Table S1). This suggests that increased contact with sites is needed to ensure they fully understand RCT study procedures. Lastly, while clinics were able to implement a pilot study involving individualised randomisation and financial incentives, a larger RCT (and hence, a larger sample size) necessitates careful consideration of whether individualised randomisation or cluster randomisation (randomisation of clinic sites) might be more feasible to avoid cross-contamination (particularly among clinics situated within the same local health district). In our case, matching clinics on key characteristics (e.g., proportion of clients who engage in daily injecting drug use) is one strategy to help ensure representativeness between study arms.

Findings from ETHOS-CM suggest high participant and clinic feasibility to implement an RCT involving financial incentives to encourage client HCV treatment initiation within drug and alcohol treatment settings. Further research is needed on how to optimise the use of contingency management interventions within drug and alcohol treatment settings to meet the varied HCV-related care needs among people who inject drugs.

## Figures and Tables

**Table 1:** Characteristics among participants enrolled in a pilot RCT involving financial incentives to initiate HCV treatment (n = 57).

	Overall	Financial Incentive	Standard of Care	
	n = 57	n = 28	n = 29	
Characteristic	N (%)	n (%)	n (%)	*p*-value *
Age, median years (IQR)	42 (37–49)	41 (37–48)	42 (37–51)	0.652
Gender, Male	36 (63%)	20 (71%)	16 (55%)	0.328
Indigenous	20 (35%)	11 (39%)	9 (31%)	0.524
Unemployed	51 (89%)	26 (92%)	25 (86%)	0.548
Homeless	8 (14%)	5 (17%)	3 (10%)	0.457
Completed Year 10	45 (78%)	21 (75%)	24 (82%)	0.747
Imprisoned in past 6 months	8 (14%)	4 (14%)	4 (13%)	0.612
Injected daily last month	21 (36%)	14 (50%)	7 (24%)	0.022
Main drug injected last month, methamphetamine	25 (43%)	16 (57%)	9 (31%)	0.068
Self-report hazardous alcohol use (AUDIT-C)	6 (10%)	2 (7%)	4 (13%)	0.612
Currently receiving OAT	33 (57%)	13 (46%)	20 (68%)	0.100
Currently receiving OAT, buprenorphine	19 (33%)	8 (28%)	11 (37%)	0.164
Self-report ever treated for HCV	18 (31%)	10 (35%)	8 (27%)	0.509
Liver disease stage, median kPa (IQR)	5.6 (4.6–7.9)	5.2 (4.2–7.4)	6.7 (5.3–9.7)	0.025

* Pearson’s chi-squared test.

## Data Availability

The datasets presented in this article are not readily available because the data are a part of an ongoing study. Requests to access the datasets should be directed to the corresponding author.

## Supplementary Materials

The following supporting information can be downloaded at: https://www.mdpi.com/article/10.3390/v16111763/s1, Figure S1: Incentive amount received at each endpoint by study arm; Figure S2: Participant flow diagram; Table S1. Sensitivity analysis of HCV treatment outcomes by study arm excluding the one site with a protocol deviation; Table S2. HCV care cascade post-diagnosis in a pilot RCT involving financial incentives to initiate HCV treatment; Table S3. HCV treatment outcomes at 12 weeks post-enrolment by study arm.
