# Supplementary material for: A Pilot Randomised Controlled Trial Involving Financial Incentives to Facilitate Hepatitis C Treatment Uptake Among People Who Inject Drugs: ETHOS Engage Study
Source: Viruses. 2024 Nov 12;16(11):1763. doi: 10.3390/v16111763 (PMC11599082; doi:10.3390/v16111763)
Supplement: Supplementary file 1 [file viruses-16-01763-s001.zip › viruses-3213306-supplementary.pdf]

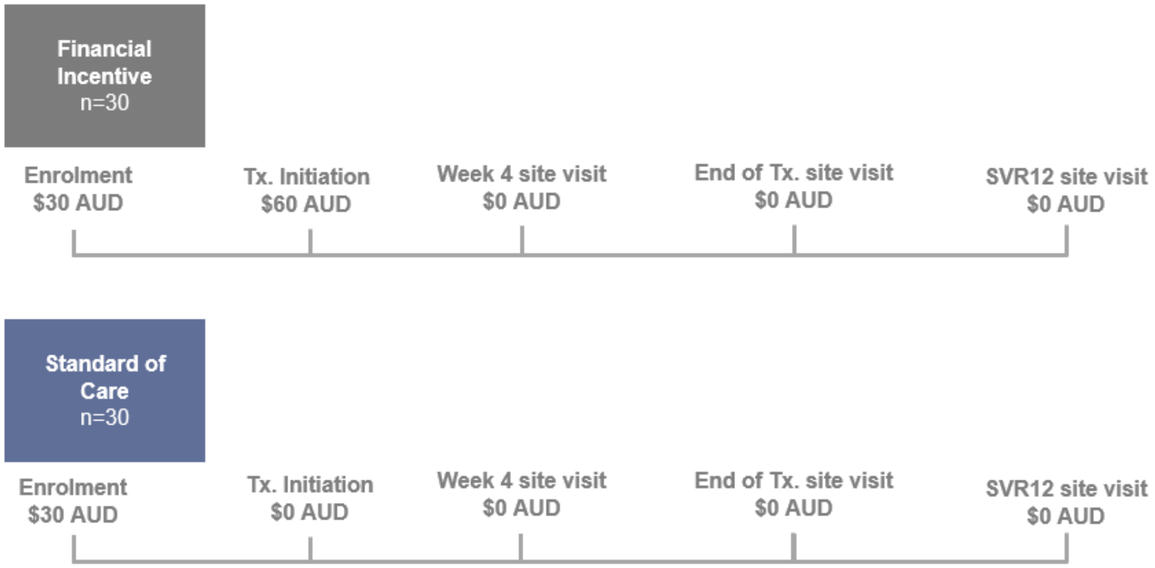

Supplementary Figure S1. Incentive amount received at each endpoint by study arm (n=60)

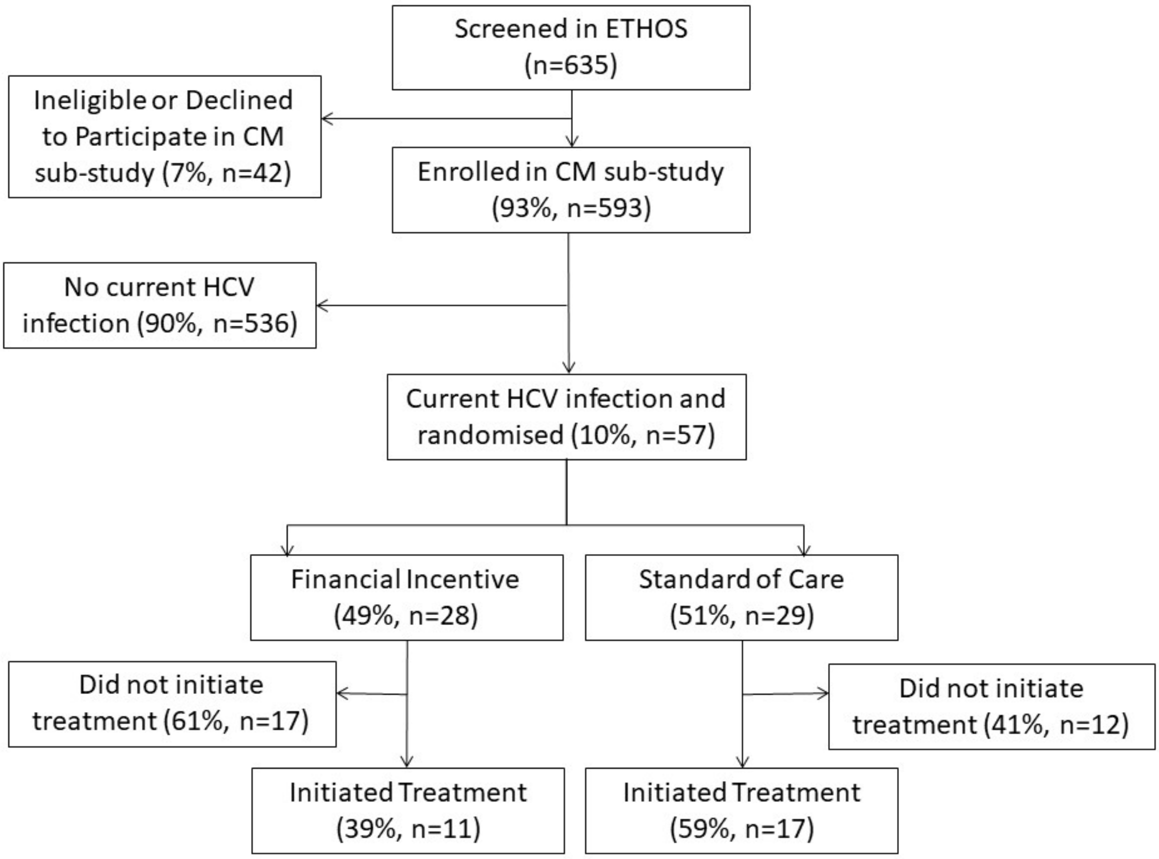

Supplementary Figure S2. Participant flow diagram (n=635)

Supplementary Table S1. HCV care cascade post-diagnosis in a pilot RCT involving financial incentives to initiate HCV treatment (n=57)

		Overall n=57	Financial Incentive n=28	Standard of Care n=29
Initiated treatment		27	11	17
Treatment completion	Yes	21	8	14
	No	2	1	1
	Unknown	4	2	2
End of treatment response	Yes	6	3	4
	No	6	1	5
	Unknown	9	4	5
SVR12	Yes	12	6	6
	No	2	1	1
	Unknown	7	1	7

Supplementary Table S2. HCV treatment outcomes at 12 weeks post-enrolment by study arm (n=57)

	Overall	Financial Incentive	Standard of care	p value*
Total (n)	57	28	29	
Treatment initiation (% of all)	28 (49%)	11 (39%)	17 (59%)	0.144
Treatment completion (ITT- % of initiated treatment)	22 (79%)	8 (73%)	14 (82%)	0.832
End of treatment response (ITT- % of initiated treatment)	7 (25%)	3 (38%)	4 (24%)	0.634
SVR12 (ITT- % of initiated treatment)<br>SVR12 (mITT**- % of SVR12 result)	12 (43%)<br>12 (100%)	6 (55%)<br>6 (100%)	6 (35%)<br>6 (100%)	0.338

*Pearson's chi-squared test

**mITT: proportion of participants who were tested at 12 weeks post-treatment who were HCV RNA negative

Supplementary Table S3. Sensitivity analysis of HCV treatment outcomes by study arm excluding the one site with a protocol deviation (n=51)

	Overall	Financial Incentive	Standard of care	p value*
Total (n)	51	22	29	
Treatment initiation (% of all)	26 (51%)	9 (41%)	17 (59%)	0.210
Treatment completion (ITT- % of initiated treatment)	20 (77%)	6 (67%)	14 (82%)	0.665
End of treatment response (ITT- % of initiated treatment)	6 (23%)	2 (22%)	4 (24%)	0.679
SVR12 (ITT- % of initiated treatment)<br>SVR12 (mITT**- % of SVR12 result)	11 (42%)<br>11 (100%)	5 (56%)<br>5 (100%)	6 (35%)<br>6 (100%)	0.164

*Pearson's chi-squared test

**mITT: proportion of participants who were tested at 12 weeks post-treatment who were HCV RNA negative
